# Depression in an intercultural psychotherapeutic context

**DOI:** 10.1192/j.eurpsy.2025.49

**Published:** 2025-08-26

**Authors:** E. Sönmez Güngör

**Affiliations:** Psychiatry, Erenköy Mental Health and Neurological Diseases Training and Research Hospital, Istanbul, Türkiye

## Abstract

**Abstract:**

Depression is widespread globally and is expected to be the leading cause of disability by 2030, according to the Global Burden of Disease study. Psychosocial factors, such as gender, life events, and migration experiences, increase the risk of depression, with migrants and asylum seekers showing particularly high depression rates. However, cultural differences and social background of individuals are often not adequately addressed in medical settings, which lead to misdiagnoses and mismanagement. Language and cultural barriers may complicate communication of subjective experiences, which consitute a critical part of the expression of symptomatology in depression. Depressed patients may have challenges in verbal expression of their symptoms and distress may be communicated through somatic symtoms, or what might be called as “organ language” or “somatic idiom”, especially in the context of migration. Indeed, somatization is closely linked to the experience of migration stress and can be a form of cultural adaptation. Moreover, the degree of somatization in migrants is found to be related to their level of integration into the host culture. Therefore, the diagnosis and management of depression in the intercultural context can be challenging, as somatic complaints may obscure the recognition of underlying psychological distress. This can delay the seeking of psychiatric help, as patients are often reluctant to attribute their symptoms to psychological causes. Fear of stigma, or perceiving the patient as overly-dependent or attention-seeking might hinder processes of treatment and care. Existing treatment modalities with highest level of evidence, such as CBT, should be culturally adapted. Cultural adaptations involve modifying language, metaphors, and treatment goals to resonate with the patient’s cultural context. While there is some evidence on the positive impact of culturally-adapted interventions including CBT, more research is needed to refine these approaches and address gaps in the literature. Psychotherapy in the intercultural context requires both cultural competence and time for dialogue.

**Disclosure of Interest:**

None Declared

